# Perforating Ulcer of the Appendix Vermiformis

**Published:** 1844-02-10

**Authors:** 


PERFORATING ULCER OF THE APPENDIX VERMIFORMIS.

Dr. Lees laid before the Dublin Pathological Society some recent specimens of disease of the intestines in children. The first were from a child four years old, that had died during this week in the South Union Hospital. This child had been suffering from chronic diarrhœa, and was in a state of great emaciation; the discharges from the bowels were intermixed with shreds of lymph in the membranous form, but there were no sanguineous dejections; there had been incessant vomiting during two days previous to death, the large intestine was very vascular, and all its coats thickened; beneath the mucous membrane were several ecchymosed spots; the vascularity was greatest in the rectum; the lower portion of the ileum also was very vascular, thickened and coated internally with adherent lymph; the coats of the stomach were thickened, and there was a deposition of lymph on its mucous surface, similar to that on the ileum; the mucous membrane itself was of a brown color, and very vascular, presenting an appearance similar to that called *mammillary* by Louis, and *granular* by Hodgkin. From the pylorus to the ileum the intestine had no unhealthy appearance.

The next specimens were from a child of fifteen months old, which had been admitted only a week previously, at which time it was described to have been laboring under chronic diarrhœa. On examination of the body after death, the mesenteric glands were found affected by tubercle; the mucous surface of the large intestine was thick, warty, and vascular; but the most remarkable lesion was in the appendix vermiformis, about the middle of which was observed a large patch of green lymph, which covered an ulcer that had nearly perforated into the peritoneal cavity; the mucous membrane appeared to have been removed round a large portion of the tube of the appendix, and in the centre of this ulcerated part was the minute perforation, which was only prevented from communicating with the sac of the peritoneum by this deposit of bright green lymph; there was no peritonitis. Dr. Lees said that this case was interesting, on account of the early age of the subject, as, although many cases are on record, and the pathological lesion has been fully described, yet, the earliest age hitherto mentioned at which it had occurred, was six years, in a case described by Dr. Burne, in his memoir on tuphloenteritis, published in the "Medico-Chirurgical Transactions."—*Dublin Journ. Med. Sci.*, November, 1843.

